# Power and Powerlessness in a Group Based Digital Story Telling Project-An Exploration of Community Perceptions of Health Concerns in Urban Malawi

**DOI:** 10.3389/fpubh.2022.826428

**Published:** 2022-04-22

**Authors:** Deborah Nyirenda, Chipiliro Payesa, Jolly Ntaba, Rachel Mhango, Patricia Kingori, Michael Parker, Nicola Desmond

**Affiliations:** ^1^Malawi Liverpool Wellcome Trust Clinical Research Programme, Blantyre, Malawi; ^2^Malawi Universities of Business and Applied Sciences, Blantyre, Malawi; ^3^The Ethox Centre, Wellcome Centre for Ethics and Humanities, University of Oxford, Oxford, United Kingdom; ^4^Liverpool School of Tropical Medicine, Liverpool, United Kingdom

**Keywords:** Digital Story Telling, participatory visual method, Africa, public engagement, power and powerlessness, WASH

## Abstract

Digital Story Telling (DST) is an art-based research method used to explore embodied experience of health and initiate dialogue with under-represented groups on issues affecting them. It involves engaging participants to create and share their stories using photos, drawings, and audio recordings in short videos. Benefits of DST include enhancing co-creation of knowledge, empowering participants to confront dominant narratives and revise inaccurate representations. We report our experiences and reflections of using DST to explore community perceptions of health concerns in urban Malawi. Community leaders were briefed about the project before and after study related activities. Three participatory workshops were organized to train community members in DST, support them to develop videos and discuss their experiences of DST. Twenty-six participants from two high density urban communities consented to be part of the workshops. They were all new to DST. All the 26 participants were invited together to the three workshops and their DSTs were developed in smaller groups (*n* = 7), based on their geographical location. Although we engaged residents from selected communities to share priority health concerns, all the seven groups presented challenges pertaining to Water, Sanitation and Hygiene (WASH), and their powerlessness to address the complex challenges. The collective focus on WASH showed that DST effectively empowered communities to present priority health concerns. The inability of community members to address the challenges without external assistance or failure to use findings from DST to generate social change however raise questions on the ideals of empowerment and social justice. In addition, lack of financial resources or technical know-how to produce digital stories and unequal power relationships between service providers and community, may affect the use of DST for community activism among socio-economically disadvantaged groups. We conclude that DST empowered participants to articulate genuine health challenges that they felt powerless to address. We question the realization of “empowerment” and social justice of vulnerable participants in cases where structural challenges present obstacles to effectively address social inequalities.

## Background

Reducing social inequalities is one of the most important ways of ensuring social justice and improving health. Inequalities in power and resources at global, national, and local levels continue to contribute to health inequities between rich and poor populations. A key component in effective interventions aimed to address health inequities is engagement with affected communities. Top-down bio-medical research and interventions aimed at improving public health have been critiqued for producing knowledge from the perspective of powerful outsiders, inadequately informed by the representations, insights and values of community members (1). Considering that the major determinants of health are social in nature, participatory approaches of engaging communities are promoted to identify and address social conditions that lead to diseases.

Community engagement is defined as ‘a process of working collaboratively with and through groups of people affiliated by geographic proximity, special interest, or similar situations to address issues affecting the wellbeing of those people’ (2). The main purpose of introducing participatory approaches of engaging communities is to empower communities to identify solutions to their challenges, with limited external influence from outsiders (3). Thus, community engagement aims to empower local people to develop a critical consciousness and to determine the best actions to improve their lives. While global health research has been critiqued for privileging the voices of experts and powerful actors, community engagement was introduced to incorporate locally defined priorities and perspectives. In addition, community engagement is also widely promoted in the conduct of health research and interventions to enhance the relevance of projects, address health inequities and ensure social justice (4–6).

Several publications have emphasized the need for community engagement in medical research (7–10), but few studies have used participatory approaches to explore community priority health concerns or experiences of community engagement in Malawi. This study aimed to assess if Digital Story Telling (DST) could be used as a tool to explore community perspectives of health concerns and community engagement approaches or participatory health interventions to allow communities to help shape the terms of engagement with researchers. In addition, we intended to explore if DST can be used as an evaluation tool for community engagement practices to enable us to generate evidence on culturally relevant approaches in urban contexts.

Digital Story Telling is an art based participatory research used to engage vulnerable or under-represented population groups to address health inequities. Participants for DST are engaged in a collaborative process to articulate their own meanings and experiences of health by synthesizing digital photos and audio recordings to present digital stories (11, 12). Thus, DST positions participants as “experts” and allows them to step into positions of power to create and share their lived experiences of disease or health interventions. Since participants take the lead to discuss issues that concern them, this approach is seen as appropriate for marginalized populations because it allows self-representation of a story “from the inside out” and avoids imposition of researcher or “outsider” views of the community (13). This approach also empowers participants to confront or resist dominant narratives and to revise inaccurate representations by using visually appealing accounts. Several studies have shown that DST is acceptable to vulnerable groups, empowering to participants whose voices are rarely heard and that it promotes positive behavior change (11–15). Allowing participants to share their own stories supported by digital photos is also perceived as engaging and relevant to present day technology, as well as visual culture (16). Thus, findings from DST are likely to lead to social justice because they are understood and applied by the general public including low literate groups, rather than text based research outcomes which are predominantly understood by academic readership (13). While many DST projects in health research have focused on individual experiences, we report our experiences of group-based DST with selected participants from urban communities where many health research projects and interventions continue to be conducted.

We draw from Gaventa's theories of power and participation to discuss power dynamics and mobilization of bias in participatory processes. According to Gaventa (17), power involves the capacity of A (individual or groups) to prevail over B in decision making or shaping B's actions about a situation. If the interests of A and B are different, A can potentially exercise power to put barriers around decision making spaces and thereby maintain the quiescence of B and mobilization of bias. Mobilization of bias refers to a dimension in the exercise of power where institutions or knowledge frameworks admit some issues while excluding others (17). The capacity of A to influence which issues to include or exclude in decision making accords A more power which may potentially affect B's response to remain quiescent. Thus, power and powerlessness may re-enforce each other, leading to further inequality which may be difficult to alter unless B acts to overcome A's power.

## Methodology

### Setting

Malawi is one of the poorest countries in the world with an estimated population of 19 million; 69% of the population live below the international poverty line of < $1.90 a day (18). Both the adult and under five mortality rates are among the highest in the world, at 254 and 39 per 1,000 population, respectively (18, 19). Twenty-eight per 100,000 of these deaths are attributed to unsafe water, unsafe sanitation and poor hygiene (18). While statistics show that 17% of the population in Malawi reside in urban settings, a majority of the urban residents (65%) live in urban slums (18).

The DST project was conducted with participants from high density urban locations: Bangwe and Ndirande in Blantyre, the second largest city in Malawi. Due to urbanization, most of the residents have migrated to the city to seek employment or business opportunities. As such, residents in the city have multi-ethnic backgrounds, different traditional beliefs and are socially loosely knit compared to rural communities. Both Bangwe and Ndirande townships are quite similar in terms of socio-demographic characteristics and social organization. Most of the urban poor are faced with poverty, food insecurity, poor sanitation, and hygiene. For instance, recent studies conducted in both townships revealed that most of the communal water points were highly contaminated and not safe for consumption (20, 21). Due to high prevalence of diseases such as diarrhea, tuberculosis malaria and HIV/AIDS, several health research projects, and participatory health interventions are conducted in these settings. We chose Bangwe and Ndirande as study sites because residents have been exposed over time to community-based health research projects and interventions on HIV self-testing, tuberculosis, malaria, typhoid vaccine trials and several others.

Both Bangwe and Ndirande fall within Blantyre city council which is mandated to offer services in relation to waste management, communicable disease control and other public amenities. Bangwe and Ndirande are divided into blocks headed by chiefs and sub chiefs on the traditional administrative level while ward councilors operate on the legislative level. The traditional leaders' positions are often nominated by the chieftaincy clan, and they represent communities during meetings with service providers and settle minor disputes. Councilors on the other hand are elected by residents to represent community concerns at the city council and to ensure that relevant services are provided.

### Digital Story Telling Research Approach

**Figure 1:**
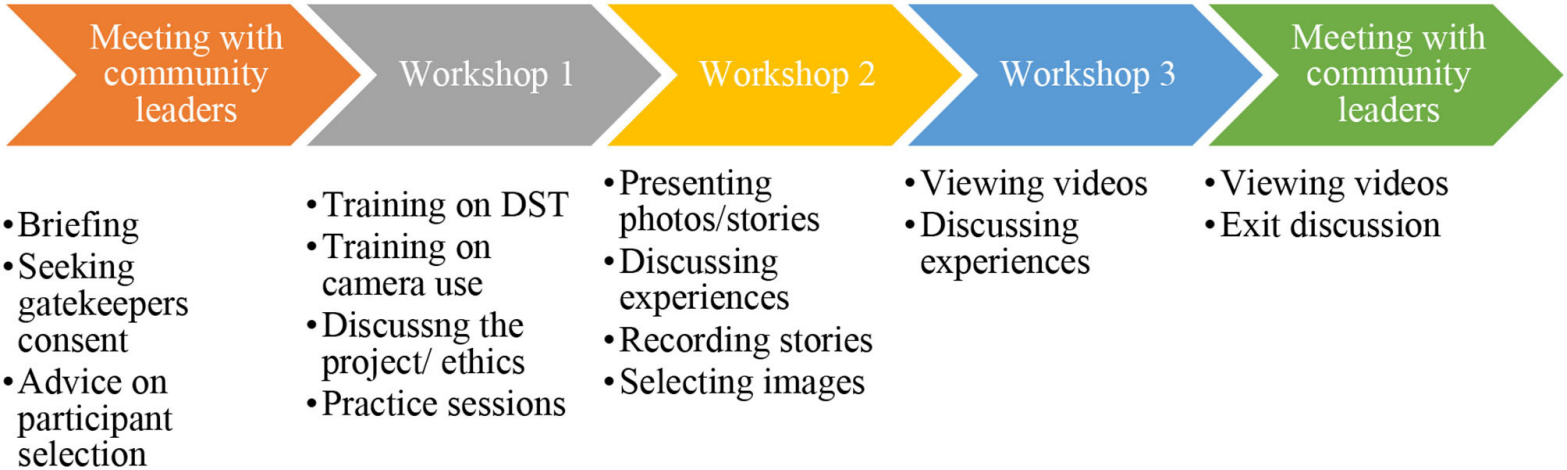
Digital Story Telling workshops.

We used DST because it allows self-representation of experiences visually through photos and participants own voices. We planned to have three participatory workshops with participants from urban communities in Bangwe and Ndirande townships (see Figure 1). In this paper, we define community as geographical settings or blocks where DST participants resided.

Prior to the workshops, we had two meetings with community leaders to discuss the study, seek gatekeepers' consent, and consult them on the most relevant approach to identify participants for the participatory workshops. Our plan was to purposively select residents from various community groups, based on age, gender as well as knowledge and experiences of medical research and health interventions to ensure that they express their views about community engagement and health concerns. The community leaders helped to map all community groups in their geographical locations. They also suggested an additional criterion of ensuring that selected participants came from all the geographical locations headed by block leaders, and we took this into consideration. With the help of the community leaders and community advisory group members, we briefed various community groups such as village health committees, women's health committees, youth groups as well as groups of people living with disabilities about the project and collected their contact details. Twenty-six participants were purposively selected from various community groups and contacted by telephone to invite them to the workshops. Text messages were also sent to participants to remind them about the workshop.

All the twenty-six participants (12 men and 14 women) attended the workshops (see Table 1 for socio demographic details). Most of the participants had secondary education, and all were between the ages of 20–50 as shown in Table 1. The workshops were conducted between November 2020 to March 2021 when COVID-19 cases declined and restrictions on public gatherings were relaxed by the Ministry of Health. All COVID-19 preventive measures were observed, participants were encouraged to wear face masks, observe social distance, and sanitize their hands where necessary. All workshop participants were compensated with $10 for attending each workshop, in line with regulations from the local ethics review board. The participants were also served with snacks and drinks during workshops. The research team included two Social Scientists (DN and CP) and two DST and media engagement professionals (JN and RM).

**Table 1:** Sociodemographic details of workshop participants.

	**Bangwe**	**Ndirande**	**Total**
**Gender**			
Female	8	6	14
Male	4	8	12
**Age**			
20–30	5	6	11
31–40	3	3	6
41–50	3	4	7
51–60	1	1	2
**Education**			
Primary	1	2	3
Secondary	11	12	23
Tertiary	0	0	0

### Ethics Approval

The study was approved by the University of Malawi, College of Medicine Research Ethics Committee (P.01/20/2911) and Liverpool School of Tropical Medicine Research Ethics Committee (20-001). All potential participants were contacted by phone to inform them about the project and to give them more time to consider their participation at the workshops. Written consent was sought on an individual basis from all participants prior to each workshop. We also sought approval from the participants and community leaders to share the videos and experiences with other stakeholders.

#### Participatory Workshop One

At the first workshop, we presented the project and sought written consent from participants who were interested to participate. Thereafter, we oriented participants to DST, ethics of DST, how to use digital cameras and finally, discussed prompt questions to guide them to develop scripts for their stories. The prompt questions focused on the themes of community concerns, health interventions aimed to address community concerns, perspectives of successes and challenges of community engagement approaches used, perspectives on how they wish to be engaged and finally local ethical issues that service providers must be aware of (see Appendix 1, for list of topics that were covered). Participants demonstrated that they understood the prompt questions from the discussions. Most of the participants were new to each other, except for few who knew each other from their respective places of residence. Team building exercises were used throughout the workshop to build rapport and encourage team bonding. Towards the end of the workshop, the 26 participants were divided into seven groups based on place of residence and their DSTs were developed in seven smaller groups. The participants were split into these groups to encourage participation of individuals who were initially not comfortable to use the cameras and to allow them to discuss community health concerns, experiences of community engagement or participatory health interventions. Four women and three men were selected as group leaders by group members based on their own assessment of individual strengths. Each group (*n* = 7) was given a camera to practice telling a story using digital photos. We observed participants in their small groups as they practiced taking photos with the cameras and present their digital stories to the whole group. We did not identify major technical issues or challenges pertaining to gender dynamics that affected participation. Older participants were however reluctant to use the cameras compared to the young ones. Thereafter, we agreed on the timelines for the participants to develop their stories and take photos in their respective communities before the next workshop. The group leaders went home with the cameras and worked in their respective groups to take pictures about their story for 2 weeks. Phone calls were made to group leaders to check on their progress and to invite them for the second workshop.

#### Participatory Workshop Two

All the 26 participants attended the second workshop where they presented their stories and pictures. Written consent was again obtained from all, prior to the workshop discussions. Representatives from all the seven groups were asked to present their pictures without narrating their story. After showing pictures, the group representative was then asked to explain their story to the audience. To our surprise, we noted that the theme for all the seven groups was about poor hygiene, unsafe water, and sanitation as their main health concern. None of the group members focused on their experiences of community engagement, participatory health interventions and other issues that were included in the prompt questions. Thereafter, each group was asked to finalize their scripts and select one person to narrate their story in the local language for audio recording. We also worked with each group to select images to explain their stories because some of the groups had captured too many pictures while others had captured a few. Thereafter our film makers assisted to align the voice over narrations and pictures to produce short video clips. All the participatory workshops ran smoothly except for a few technical issues pertaining to cameras.

#### Participatory Workshop Three

We invited all the participants to the third workshop to view the videos and engage them in a discussion about their experiences of being involved in the project. Twenty-five participants attended the workshop and only one participant had traveled out of town. We showed the seven videos to the group and invited them to discuss their experiences of being involved in the project, their views about the videos and why they all focused on one theme. Thereafter, we organized two meetings with community leaders at each site where we showed and discussed the videos. We also informed the community leaders that we had completed the project and thanked them for allowing us to work in their communities. Workshop discussions were audio recorded and documented in field notes.

## Findings/Results and Discussion

Our discussion will focus on two broad themes of (a) power and powerlessness in group-based DST and (b) ethical and practical challenges of group-based DST. While many DST projects in health research have focused on individual experiences, we planned to use DST to explore community's health concerns, group experiences of community engagement and participatory health interventions. The benefits of this approach were that it encouraged participation and contribution from individuals who felt less technologically competent, and it empowered them to reflect on genuine issues of concern to their community. Participants focused their digital stories on Water, Sanitation and Hygiene problems rather than other health issues or experiences of community engagement as intended by the researchers. On the other hand, participants raised concerns pertaining to their safety that could impact on processes and outcomes of community-based DST projects.

### Power and Powerlessness in Group Based Digital Story Telling

**Figure 2:**
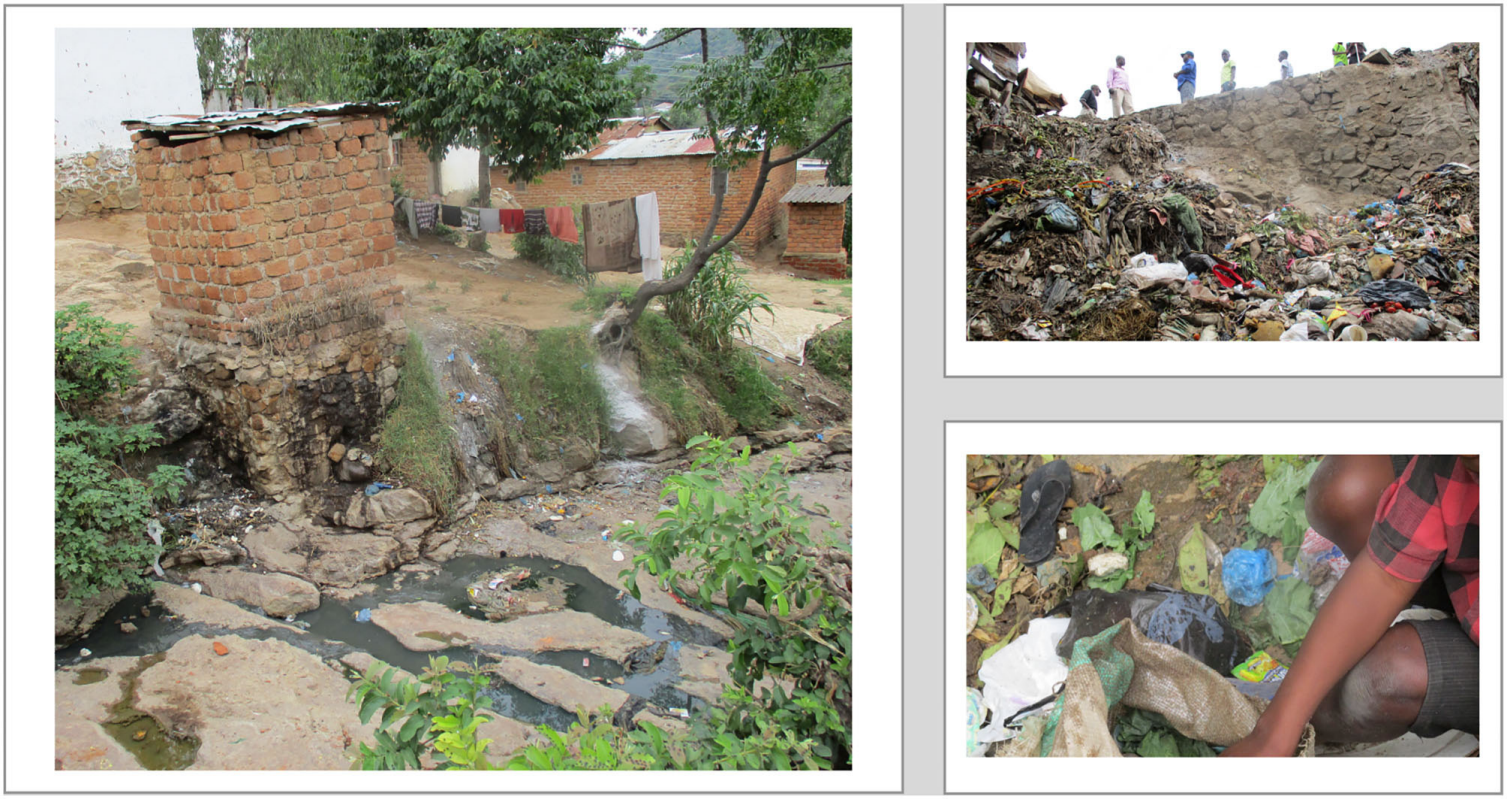
Selected photos captured by DST participants.

The group-based DST project empowered participants to raise genuine health concerns that affected their communities and exposed them to high risk of infectious diseases (see Figure 2). At the third workshop, we engaged participants to discuss why their focus was on problems related to WASH rather than their experiences of community engagement and other health concerns. They indicated that existing interventions already focused on addressing present social problems and managing diseases such as HIV/AIDS, TB, and malaria. Such diseases or challenges also affected a smaller proportion of the community. As such, experiences with such interventions could only be expressed by the community affected with a particular disease. The challenges in relation to WASH, on the other hand were a shared problem because they affected every community member and were more visible, yet they received less attention from service providers and other powerful actors. Poor hygiene, sanitation and unsafe water was also seen as the main cause of ill health and hence an important problem that needed to be addressed.

In addition, participants indicated that most community members were not often involved in participatory processes to voice out their health concerns and they were usually not consulted on health interventions to deliver to the community, except for few community leaders or community representatives. The participants indicated that communities were usually informed when an intervention is ready to be implemented. As such, they could not speak collectively about their experiences of community engagement or participatory health interventions. The lack of attention on community engagement or participatory health interventions could be a reflection that our participant group was not homogenous to speak collectively on community engagement. On the other hand, their focus on WASH demonstrated that the DST effectively empowered them to discuss priority health concerns that affected them as a collective.

In as much as the DST project empowered participants to articulate genuine health concerns, it also created a platform for participants to express their sense of powerlessness to address structural challenges leading to WASH. During the third workshop, we asked participants to suggest ways of addressing challenges identified in the videos. They stated that challenges related to poor refuse and sewage disposal as well as unsafe drinking water were beyond their control; thus required the attention of powerful actors to improve the sewage system, provide refuse skips and increase water points. Similarly, community leaders felt powerless to address the WASH challenges. They highlighted that previous attempts to educate communities about the importance of good hygiene and introduce penalties for non-compliance to health interventions had not been successful. Regular victims of the penalties were usually the most vulnerable households who could not genuinely afford to have pit latrines, rubbish pits, or afford the penalties for not having them. In addition, most of the refuse was dumped in inappropriate places at night, thereby making it difficult to catch the culprits. As such, they asked for assistance from service providers to provide safe water, skip bins and ensure timely collection and disposal of waste. The community leaders also indicated that community members were used to receiving payments and other handouts from politicians and other service providers for rendering community services. This made it difficult for the community leaders to engage community members to do any voluntary service to clean the streets or markets without any form of payment. The practices of giving handouts for community services and the lack of attention to WASH challenges by powerful actors over time may have led communities to psychologically adapt to a sense of being powerless and re-enforced views of quiescence.

### Ethical and Practical Dilemmas of Group-Based DST

#### Ethical Obligation to Address Challenges Identified in DST

The request from workshop participants and community leaders for academic researchers to address the challenges they identified raise important questions about researchers' ethical obligations to respond to community needs and ensure social justice. Even though the DST enabled participants to voice their concerns, they could not identify immediate solutions to the challenges. On the other hand, failure to use findings from DST to generate social change raise questions on the social value of DST to community participants. The problems raised by DST participants were well-known and visible to powerful actors who were mandated to improve WASH. As academic researchers, we faced dilemmas to use the digital stories to engage powerful actors on behalf of the community because they were already aware, and it implied that community voices can only be heard if other powerful actors intervene. This also implies that communities will remain disempowered if they must depend on powerful actors to support them with approaches such as DST to address inequalities. In addition, Gaventa (17) argues that to address power inequalities, the powerless groups must act to overcome the effects of power and being powerless. As such, DST could be used by participants for community activism to demand services from relevant service providers aimed to address WASH problems.

#### The Feasibility of Using DST With the Most Vulnerable Groups

Even though DST promises to be a powerful tool for community activism, we questioned the feasibility of using DST as a tool for community activism to allow most vulnerable groups to speak to power. Ideally, empowering communities should aim to equip communities to identify their problems and address them with minimal external assistance. Our experiences however showed that conducting DST projects required human, financial, and material resources to procure cameras, train participants on DST, camera use and produce videos. Even though many residents in townships had access to smart phones with camera, the technical-know how to present their stories live, using a projector to accompany their live voice over and the editing process for the packaged stories was a challenge. Many were handling the camera for the first time and needed more time for training in picture compositing, logical sequencing of telling a cohesive story and editing, which had financial implications. As such, we felt most vulnerable community groups may not be able to organize DST projects on their own without external assistance due to lack of human and financial resources. The fact that less powerful actors must depend on powerful actors like academic researchers to have digital stories produced therefore raise questions on the ideals of empowerment and whether DST is a viable option to engage communities in solving their problems.

#### Potential Risks to DST Participants

Participants main concerns about the community-based DST project pertained to physical and verbal assault from fellow community members for taking photos of their plight. We asked participants at the first workshop to reflect on challenges that they might experience in their respective communities. Workshop participants presented threats of physical assault from fellow community members if they see them taking pictures or demands for money in exchange for taking their photos. We introduced participants to ethics of DST and consenting processes for taking photos of other community members, but participants avoided photos of fellow community members. We assumed that engaging residents as participants was a way of leveraging their local expertise and that they would be trusted by fellow community members; but they indicated that their embeddedness in the community posed risks in this urban community. Participants who shared this view stated that fellow community members may react because they did not benefit financially while participants of the DST benefited financially from workshop allowances. As researchers, we felt this was a potential limitation for community-based DST because the fear of physical assault for taking photos could potentially lead participants to focus on photos about their physical environment and hence impact on the output of the project. Furthermore, fear of reprisal for speaking to power may also potentially limit participants to focus on issues that were easy to tell and leave out sensitive topics. Some community leaders expressed uneasiness that the videos had selectively focused on negative aspects of their communities in relation to WASH while leaving out other positive stories. While they admitted that the videos reflected their lived reality, they also expressed despair about the magnitude of sanitation challenges presented in their respective areas. This presented another dilemma on whether to disseminate outputs from DST that may cause discomfort to community residents. Apart from safeguarding confidentiality and anonymity of participants, it is important to carefully consider how to prevent discomfort and othering.

## Conclusion

In conclusion, this paper reports our experiences of using Digital Story Telling to explore community's health concerns in an urban setting in Malawi. We have shown that DST empowered participants to articulate community health concerns and their powerlessness to address structural challenges that were deeply ingrained. Since DST allowed community members to express locally defined health priorities, it can potentially support priority setting for health research, interventions, and co-production of knowledge.

While engaging disadvantaged groups in participatory processes as co-producers of knowledge empowers them to transform their situation (22); our experiences demonstrate that DST empowered participants to express their priority health concerns as well as their powerlessness to address the complex challenges. Community members felt powerless because structural challenges pertaining to WASH were aggravated by urbanization, overpopulation, and poverty; and therefore, require long term multi sectoral approaches. Unequal power relations between communities and service providers in priority setting for health research/interventions may also have led to mobilization of bias where priority problems affecting communities were not considered for interventions.

Though existing literature shows that DST promotes decolonization of knowledge production and minimizes imposition of outsiders views (13); unequal power relationships can potentially lead powerless groups to be more dependent on powerful actors and not critically reflect on alternative solutions. Rather than exploiting the spaces of participation to critique power, reverse dominant narratives or inaccurate representations, unequal power relations may still lead participants to reproduce or re-enforce the dominant narrative that problematise them. We question the realization of “empowerment” in cases where community members must rely on outsiders to amplify their voices through techniques such as DST and existing structural challenges present obstacles to address social inequalities. The inability to immediately respond to the challenges also present an ethical dilemma on social justice.

## Main Points in the Paper

- DST offered an opportunity to participants to raise genuine health concerns that exposed communities to high risk of infectious diseases, as well as obstacles to effectively address the challenges.
- Community's inability to address the community health concerns and dependency on external help raise questions on realization of empowerment as well as social justice.
- The high costs and technical expertise required to implement DST projects also raise questions on the ideals of empowerment since most vulnerable community groups may not be able to organize DST projects without external assistance.

## Data Availability Statement

The raw data supporting the conclusions of this article will be made available by the authors, without undue reservation. For the purpose of open access, the author has applied a CC BY public copyright license to any author accepted manuscript version arising from this submission.

## Ethics Statement

The studies involving human participants were reviewed and approved by University of Malawi, College of Medicine Research Ethics Committee (P.01/20/2911) and Liverpool School of Tropical Medicine Research Ethics Committee (20-001) in UK. The patients/participants provided their written informed consent to participate in this study.

## Author Contributions

DN conceptualized the research design, led the research and implementation teams, and drafted the manuscript. CP, JN, and RM were involved in implementation and contributed to the manuscript. PK, MP, and ND mentored DN and contributed to research design and manuscript. All authors contributed to the article and approved the submitted version.

## Funding

The study was funded by a post doctoral fellowship award from the Global Health Bioethics Network (GHBN). The GHBN was funded by a Wellcome Trust strategic award (096527).

## Conflict of Interest

The authors declare that the research was conducted in the absence of any commercial or financial relationships that could be construed as a potential conflict of interest.

## Publisher's Note

All claims expressed in this article are solely those of the authors and do not necessarily represent those of their affiliated organizations, or those of the publisher, the editors and the reviewers. Any product that may be evaluated in this article, or claim that may be made by its manufacturer, is not guaranteed or endorsed by the publisher.

## References

[1] Sullivan M Kone A Senturia KD Chrisman NJ Ciske SJ Krieger JW. Researcher and researched-community perspectives: toward bridging the gap. Health Educ Behav. (2001) 28:130–49. 10.1177/109019810102800202 11265825

[2] McCloskey DJ Akintobi TH Bonham A Cook J Coyne-Beasley T. Principles of Community Engagement. 2nd ed. COMMUNITY Engagem (n.d.). 197 p.

[3] Chambers R. Whose Reality Counts?: Putting the First Last. London: Intermediate Technology Publications (1997).

[4] Cyril S Smith BJ Possamai-Inesedy A Renzaho AMN. Exploring the role of community engagement in improving the health of disadvantaged populations: a systematic review. Glob Health Action. (2015) 8:1–12. 10.3402/gha.v8.29842 26689460 PMC4685976

[5] O'Mara-Eves A Brunton G Oliver S Kavanagh J Thomas J Jamal F. The effectiveness of community engagement in public health interventions for disadvantaged groups: a meta-analysis. BMC Public Health. (2015) 15:1–23. 10.1186/s12889-015-1352-y 25885588 PMC4374501

[6] Yuan M Lin H Wu H Yu M Tu J Lü Y. Community engagement in public health: a bibliometric mapping of global research. Arch Public Health. (2021) 79:6. 10.1186/s13690-021-00525-3 33436063 PMC7801880

[7] Federica F. Community involvement in biomedical research conducted in the global health context; what can be done to make it really matter? BMC Med Ethics. (2018) 19:39–47. 29945597 10.1186/s12910-018-0283-4 PMC6019999

[8] Holzer JK Ellis L Merritt MW. Why we need community engagement in medical research. J Investig Med Decker Publ. (2015) 62:851–5. 10.1097/JIM.0000000000000097 24979468 PMC4108547

[9] Nyirenda D Gooding K Sambakunsi R Mfutso-Bengo J Manda L Gordons Parker M. Strengthening ethical community engagement in contemporary Malawi. Wellcome Open Res. (2018) 3:1–7. 10.12688/wellcomeopenres.14793.1 30542663 PMC6259484

[10] Pratt B de Vries J. Community engagement in global health research that advances health equity. Bioethics. (2018) 32:454–63. 10.1111/bioe.12465 30035349

[11] DiFulvio GT Gubrium AC Fiddian-Green A Lowe SE Del Toro-Mejias LM. Digital storytelling as a narrative health promotion process: evaluation of a pilot study. Int Q Community Health Educ. (2016) 36:157–64. 10.1177/0272684X16647359 27166356

[12] Fiddian-Green A Kim S Gubrium AC Larkey LK Peterson JC. Restor(y)ing health: a conceptual model of the effects of digital storytelling. Health Promot Pract. (2019) 20:502–12. 10.1177/1524839918825130 30736703

[13] de Jager A Fogarty A Tewson A Lenette C Boydell KM. Digital storytelling in research: a systematic review. Qual Rep. (2017) 2548:2548–82. 10.46743/2160-3715/2017.297029506568

[14] Gubrium AC Hill AL Flicker S. A situated practice of ethics for participatory visual and digital methods in public health research and practice: a focus on digital storytelling. Am J Public Health. (2014) 104:1606–14. 10.2105/AJPH.2013.301310 23948015 PMC4151912

[15] Rieger KL West CH Kenny A Chooniedass R Demczuk L Mitchell KM . Digital storytelling as a method in health research: a systematic review protocol. Syst Rev. (2018) 7:41. 10.1186/s13643-018-0704-y 29506568 PMC5838876

[16] Kim W Bangerter LR Jo S Langer S Larkey L Griffin J . Feasibility and acceptability of a 3-day group-based digital storytelling workshop among caregivers of allogeneic hematopoietic cell transplantation patients: a mixed-methods approach. Biol Blood Marrow Transplant. (2019) 25:2228–33. 10.1016/j.bbmt.2019.06.030 31265918

[17] Gaventa J. Power and Powerlessness: Quiescence and Rebellion in an Appalachian valley. Urbana and Chicago: University of Illinois Press (1980).

[18] World Bank. Indicators | Data [WWW Document]. World Bank Data (2022). Available online at: https://data.worldbank.org/indicator (accessed July 3, 2022).

[19] WHO. WHO Global Water, Sanitation and Hygiene Annual Report 2019 [WWW Document]. (2020). Available online at: https://www.who.int/publications-detail-redirect/9789240013391 (accessed July 3, 2022).

[20] Kamanula JF Zambasa OJ Masamba WRL. Quality of drinking water cholera prevalence in Ndirande Township, City of Blantyre, Malawi. Phys. Chem. Earth Parts ABC. (2014) 72–5:61–7. 10.1016/j.pce.2014.09.001

[21] Mtewa TK Chauluka F Mtewa AG Banda LM Kalindekafe L. Water quality assessment of various sources in peri-urban areas of Malawi: a case of Bangwe township in Blantyre. Afr J Environ Sci Technol. (2018) 12:421–8. 10.5897/AJEST2018.2542

[22] Freire P. Pedagogy of the Oppressed. Harmondsworth: Penguin Education (1972).

