# Supplementary material for: Power and Powerlessness in a Group Based Digital Story Telling Project-An Exploration of Community Perceptions of Health Concerns in Urban Malawi
Source: Front Public Health. 2022 Apr 22;10:826428. doi: 10.3389/fpubh.2022.826428 (PMC9068996; doi:10.3389/fpubh.2022.826428)
Supplement: Supplementary file 1 [file Data_Sheet_1.docx]

**Appendix 1**

**Mawu Athu project**

**Prompt questions for the digital story telling project**

1. *Tell/show us your main concerns pertaining to health issues faced by your community*

**Mungatifotokozeleko zinthu zimene zimakudetsani nkhawa ku dela kwanu/ kapaena mavuto ukhudza umoyo omwe mukukumana nawo?**

1. *Over the past 10 years, can you tell us about any efforts by your community to address health concerns faced?*

**Mu zaka khumi zapitazi, anthu akudela kwanu achitapo chani kuti athetse mavuto a zaumoyo omwe mwafotokoza?**

1. *Over the past 10 years, can you tell us any efforts/ health interventions by other service providers to address health concerns faced?*

**Mu zaka khumi zapitazi, amabungwe, aboma kapena akafukufuku achitapo chani kuti athetse/athane ndi mavuto aza umoyo omwe mwafotokoza?**

- *Tell us the health interventions that you think have helped to address community health problems effectively?*
  - **Ndi ma project ati/ntchito/kafukufuku wanji amene mukuwona kuti wathandiza kuthetsa/kuchepetsa mavuto omwe mumakumana nawo?**
- *Tell us health interventions or research projects that you have faced challenges?*
  - **Nanga ndi a project anji/ntchito zanji/kafukufuku wanji amene mukuwona kuti wabweletsa mavuto ena?**

1. *How have you been engaged/involved by service providers in addressing the health problems that you have been facing in this community?*

**Kodi ngati anthu akudela, mwagwila bwanji ntchito ndi a mabungwe/akafukufuku pothana ndi mavuto omwe mwafotokoza?**

- *Tell us the approaches that have been effective?*
  - **Ndi njila ziti zimene agwilitsa ntchito bwino kuti anthu akumudzi atenge nawo mbali pochepetsa/pothana ndi mavutowo?**
- *Tell us the approaches that you think have not been effective?*
  - **Ndi njila ziti zimene sizinagwile bwino ntchito?**

1. *What do you think should be done to address the community health concerns?*

**Inu mukuwona kuti amabungwe/akafukufuku apange chani kuti athane ndi mavuto a zaumoyo omwe mukukumana nawo ku dela lanu?**

1. *How should communities be involved in addressing these community health problems?*

**Kodi anthu akudela kwani azitenga gawo lanji pofuna kuthana ndi mavuto omwe mukukumana nawo?**

1. *Tell us the local customs/ethics that you think service providers must follow when working with communities?*

**Ndi zinthu ziti kapena malangizo/miyambo yanji imene amabungwe/akafukufuku akuyenela kutsatila pofuna kugwila ntchito ndi anthu akudela kwanu?**
